# Frequency and significance of rare *RNF213* variants in patients with adult moyamoya disease

**DOI:** 10.1371/journal.pone.0179689

**Published:** 2017-06-15

**Authors:** Mi-Ae Jang, Jong-Won Chung, Je Young Yeon, Jong-Soo Kim, Seung Chyul Hong, Oh Young Bang, Chang-Seok Ki

**Affiliations:** 1 Department of Laboratory Medicine and Genetics, Soonchunhyang University Bucheon Hospital, Soonchunhyang University College of Medicine, Bucheon, Korea; 2 Department of Neurology, Samsung Medical Center, Sungkyunkwan University School of Medicine, Seoul, Korea; 3 Department of Neurosurgery, Samsung Medical Center, Sungkyunkwan University School of Medicine, Seoul, Korea; 4 Department of Laboratory Medicine and Genetics, Samsung Medical Center, Sungkyunkwan University School of Medicine, Seoul, Korea; CNR, ITALY

## Abstract

**Purpose:**

Moyamoya disease (MMD) is a rare cerebrovascular disorder characterized by stenosis of the internal carotid arteries with compensatory development of collateral vessels. Although a founder variant of *RNF213*, p.Arg4810Lys (c.14429G>A, rs112735431), is a major genetic risk factor for MMD in East Asians, the frequency and disease susceptibility of other variants in this gene remain largely unknown. In the present study, we investigated the association of *RNF213* variants with MMD in Korean patients and population controls.

**Methods:**

For all *RNF213* variants listed in the Human Gene Mutation Database (HGMD) as disease-causing or likely disease-causing mutations for MMD, genotyping was performed using matrix-assisted laser desorption/ionization time-of-flight mass spectrometry. Genetic data from 264 adult patients with MMD were analyzed and compared with two control populations comprised of 622 and 1,100 Korean individuals, respectively.

**Results:**

Among the 30 *RNF213* variants that were listed in the HGMD, p.Arg4810Lys was identified in 67.4% (178/264) of patients with MMD and showed a significantly higher allele frequency than in the controls, giving an odds ratio of 63.29 (95% confidence interval, 33.11–120.98) for the 622 controls and 48.55 (95% confidence interval, 31.00–76.03) for the 1100 controls. One additional variant, p.Ala5021Val (c.15062C>T, rs138130613), was identified in 0.8% (2/264) of patients; however, the allele frequencies were not significantly different from those in the controls.

**Conclusions:**

These results suggest that, in our cohort of Korean patients, the p.Arg4810Lys is the only variant that is strongly associated with MMD among the 30 *RNF213* variants listed in the HGMD.

## Introduction

Moyamoya disease (MMD; OMIM 607151) is a rare cerebrovascular condition characterized by a progressive steno-occlusive vasculopathy of the large intracranial arteries with compensatory development of collateral vessels [1]. Affected individuals often present with strokes, transient ischemic attacks, and intracerebral hemorrhage [1]. The majority of moyamoya patients are of Asian descent. The annual incidence of MMD is estimated to be 0.35–0.94 per 100,000 people in Japan [1,2], and approximately one-tenth that amount in Europe [3]. Epidemiological data have shown a high incidence and prevalence of MMD in Korea. The annual incidence has steadily increased from 1.7 to 2.3 per 100,000 people from 2007 to 2011 [4].

A strong association between *RNF213* p.Arg4810Lys (c.14429G>A, rs112735431) and increased susceptibility for MMD have been reported in previous studies [5,6]. *RNF213* p.Arg4810Lys is a major genetic risk factor in East Asian MMD patients, demonstrating a predominantly autosomal dominant inheritance pattern with reduced penetrance [6]. The frequency of the *RNF213* p.Arg4810Lys variant in East Asian patients was 73–79% [5–7]; therefore, other genetic factors contribute to the onset and progression of MMD. In addition to the *RNF213* p.Arg4810Lys variant, other *RNF213* variants have been identified in both East Asian and European patients with MMD [8–10]. However, because of the limited numbers of MMD patients used in these studies [5,6], further study in a large Korean population is necessary. Therefore, we investigated the frequency of MMD-related *RNF213* variants, including *RNF213* p.Arg4810Lys, in a cohort of Korean patients with MMD compared with control populations.

## Materials and methods

### Study subjects and *RNF213* variant list

We included patients with a diagnosis of MMD at Samsung Medical Center (a tertiary referral hospital in Seoul, Korea) between February 2013 and December 2015. A diagnosis of MMD was based on transfemoral cerebral angiogram findings demonstrating stenosis or occlusion of the terminal portion of the internal carotid artery with the formation of collateral vessels compensating for the arterial occlusion. Based on the Stop Stroke Study Trial of Org 10172 in Acute Stroke Treatment (SSS-TOAST), patients with potential sources of cardioaortic embolism, extracranial atherosclerosis with significant stenosis (≥50%) on the relevant extracranial arteries, other stroke mechanisms (coagulopathy, vasculitis, arterial dissection, etc.), or incomplete evaluations were excluded.

We selected all variants listed in the Human Gene Mutation Database (HGMD professional version of 2015.3) as disease-causing or likely disease-causing *RNF213* variant for MMD. This database provides known gene lesions responsible for human inherited diseases, which are selected on the basis of published studies in the literature [11]. A total of 30 *RNF213* variants are summarized in Table 1. Matrix-assisted laser desorption/ionization time-of-flight mass spectrometry (MALDI-TOF MS) genotyping was performed for all selected *RNF213* variants. Genomic DNA extracted from peripheral blood leukocytes were subjected to MALDI-TOF MS genotyping. Control data were obtained from the Korean reference genome database for healthy Korean subjects (http://152.99.75.168/KRGDB/). The control data consisted of that from 622 and 1,100 Korean individuals. Demographic information including sex and age for the control group were not available due to the Korean Personal Information Protection. In the final step, we compared genotype data between patients and healthy controls. This study was approved by the Institutional Review Board of Samsung Medical Center (IRB approval 2008-02-046-057).

**Table 1:** Thirty *RNF213* variants associated with moyamoya disease.

Location	Nucleotide	Amino acid	HGMD accession	dbSNP
Exon 9	c.1587_1589delCGC	p.Ala531del	CD1410268	NA
Exon 26	c.4865C>T	p.Ala1622Val	CM154727	NA
Exon 41	c.11671A>G	p.Met3891Val	CM110690	NA
Exon 42	c.11797G>A	p.Val3933Met	CM154728	NA
Exon 43	c.11884A>G	p.Asn3962Asp	CM116652	rs138615753
Exon 44	c.11990G>A	p.Cys3997Tyr	CM1410266	NA
Exon 44	c.12020C>G	p.Pro4007Arg	CM129860	NA
Exon 44	c.12037G>A	p.Asp4013Asn	CM1111820	rs397514563
Exon 44	c.12055C>T	p.Arg4019Cys	CM1410267	rs139265462
Exon 45	c.12185G>A	p.Arg4062Gln	CM116653	NA
Exon 45	c.12226A>G	p.Ile4076Val	CM1410269	NA
Exon 46	c.12391C>T	p.Arg4131Cys	CM154726	NA
Exon 48	c.12554A>C	p.Lys4185Thr	CM1414304	NA
Exon 51	c.13100A>T	p.Gln4367Leu	CM129861	NA
Exon 56	c.13699G>A	p.Val4567Met	CM110691	rs145282452
Exon 56	c.13756A>C	p.Thr4586Pro	CM129858	NA
Exon 57	c.13822C>T	p.Pro4608Ser	CM116654	NA
Exon 57	c.13891C>G	p.Leu4631Val	CM129862	NA
Exon 59	c.14195A>C	p.Lys4732Thr	CM1410270	rs148776624
Exon 59	c.14248G>A	p.Glu4750Lys	CM155454	NA
Exon 59	c.14293G>A	p.Val4765Met	CM110692	NA
Exon 60	c.14429G>A	p.Arg4810Lys	CM110689	rs112735431
Exon 62	c.14587G>A	p.Asp4863Asn	CM116647	NA
Exon 63	c.14780G>A	p.Arg4927Gln	CM155455	NA
Exon 63	c.14850G>C	p.Glu4950Asp	CM116648	rs371441113
Exon 65	c.15062C>T	p.Ala5021Val	CM116649	rs138130613
Exon 67	c.15408G>A	p.Met5136I	CM129859	rs376505157
Exon 68	c.15480C>G	p.Asp5160Glu	CM116650	NA
Exon 68	c.15487G>A	p.Val5163Ile	CM1410271	rs201733659
Exon 68	c.15527A>G	p.Glu5176Gly	CM116651	NA

Abbreviations: dbSNP, single nucleotide polymorphism database; HGMD, Human Gene Mutation Database; NA, not available.

*RNF213* reference accession number: NM_001256071.1

### *RNF213* genotyping using MALDI-TOF MS analysis

Detection of *RNF213* variants was carried out by high-throughput multiplex analysis on a Sequenom MassARRAY MALDI-TOF MS (Sequenom, San Diego, CA, USA). We designed specific primers to flank the mutation sites as well as extension primers to bind adjacent to the mutation sites using MassARRAY assay design software (S1 Table). The method involves a multiplex primary polymerase chain reaction (PCR) followed by an iPLEX reaction with a single primer that is specific for each genotype.

A primary PCR was performed by combining 1 μl of genomic DNA (10 ng μl^-1^) with 4 μl of PCR cocktail in a 384-well plate. The PCR cocktail was comprised of 875.5 μl of water, 230.4 μl of PCR buffer, 184.3 μl of 25 m_M_ MgCl_2_, 23.1 μl of dNTP mix (25 m_M_ each dNTP), 460.8 μl of primer mix (500 n_M_ each), and 46.1 μl of PCR enzyme. The plate was then subjected to cycling on a thermal cycler (Model T1 plus; Biometra, Goetingen, Germany), and the cycling conditions were as follows: 1 cycle at 94°C for 4 min, 45 cycles at 94°C for 20 s, 1 cycle at 56°C for 30 s, 1 cycle at 72°C for 1 min, and a final extension step at 72°C for 3 min. Allele discrimination reactions were carried out by adding 2 μl of iPLEX reaction mix to the dephosphorylated primary PCR reaction mix. Reactions were cycled at 94°C for 30 s, followed by 40 cycles at 94°C for 5 s, 1 cycle at 52°C for 5 s, 1 cycle at 80°C for 5 s, and 1 cycle at 72°C for 3 min. The products were spotted onto a 384-spot SpectroCHIP with a MassARRAY Nanodispenser (Sequenom) and analyzed on a MassARRAY Analyzer Compact (Sequenom). If a variant or “no call” was detected in MALDI-TOF MS genotyping, the corresponding exon and intron regions of the genomic DNA were sequenced to confirm the status.

### DNA sequencing for *RNF213* variants

Targeted coding exons and flanking introns of *RNF213* were amplified using primer sets designed by the authors. Genomic DNA was extracted from peripheral blood leukocytes using standard protocols. PCR was performed on a Thermal Cycler 9700 (Applied Biosystems, Foster City, CA, USA). The PCR products were sequenced on an ABI Prism 3730*xl* genetic Analyzer (Applied Biosystems) using the BigDye Terminator Cycle Sequencing Ready Reaction Kit (Applied Biosystems). Obtained sequences were compared with the reference sequence for *RNF213* (NM_001256071.1). All mutations and their putative effects at the protein level were renamed according to the Human Genome Variation Society guidelines.

### Statistics

To assess the data quality of *RNF213* variants, *P*-values were calculated by an exact test for Hardy-Weinberg equilibrium. To analyze genotype and allele frequency data, differences between the control and disease groups were compared by a χ^2^ or Fisher’s exact test as appropriate. The calculations were performed with VassarStats (http://vassarstats.net/), and 95% confidence intervals were calculated for each value. All *P*-values were based on two-sided comparisons, and *P*-values < 0.05 were considered statistically significant.

## Results

### Characteristics of the study population

Two hundred sixty-four patients with MMD were included in this study: 185 (70.1%) were female, and the average age was 44.4 ± 14.2 years (range: 18–81 years) (Table 2). One hundred sixty-two patients (61.4%) showed bilateral involvement of the internal carotid arteries on transfemoral cerebral angiogram, and 102 (38.6%) patients showed unilateral involvement. Eighty-four (31.8%) patients had ischemic stroke, 55 (20.8%) patients had TIA, 17 (6.4%) patients had hemorrhagic stroke, and 108 (40.9%) patients were asymptomatic (Table 2).

**Table 2:** Demographic characteristics of 264 Korean patients with MMD.

Variable	No. (%) of patients
Average age in years (range)	44.4 (18–81)
Sex	
Men	79 (29.9)
Women	185 (70.1)
Laterality	
Bilateral	162 (61.4)
Unilateral	102 (38.6)
Symptoms at onset	
Ischemic stroke	84 (31.8)
Transient ischemic attack	55 (20.8)
Hemorrhage	17 (6.4)
Asymptomatic	108 (40.9)

### *RNF213* genotype and allele frequencies in control and patient groups

Genotype distributions of *RNF213* variants were in Hardy-Weinberg equilibrium. To investigate the associations between *RNF213* variants and MMD, genotype and allele frequencies were analyzed using samples from 264 Korean patients with MMD and two sets of controls comprised of 622 and 1100 individuals, respectively.

The genotype and allele frequencies at the 30 *RNF213* variant loci are listed in Table 3. *RNF213* p.Arg4810Lys was identified in 67.4% (178/264) of patients with MMD. The allele frequency of *RNF213* p.Arg4810Lys was significantly higher in MMD patients than in the 622 controls (33.90% in the MMD group versus 0.80% in the control group; *P*<0.0001) and in the 1100 controls (33.90% in the MMD group versus 1.05% in the control group; *P*<0.0001). The odds ratio for an association between the *RNF213* p.Arg4810Lys variant allele and MMD was 63.29 (95% confidence interval, 33.11–120.98) in the 622 controls and 48.55 (95% confidence interval, 31.00–76.03) in the 1100 controls. The p.Ala5021Val (c.15062C>T, rs138130613) *RNF213* missense variant was identified in 2 patients with MMD (0.8%, 2/264); however, the allele frequency was not significantly different compared to that of the control group (0.38% in the MMD group versus 0.24% in control group; *P* = 1.00). No other *RNF213* variant was identified in patients with MMD.

**Table 3:** Allele frequencies of 30 *RNF213* variants in Korean patients with moyamoya disease and two population controls.

	*RNF213* genotype			Sample size	MMD patients (*N* = 264)		Controls* (*N* = 622)	Controls* (*N* = 1100)	*P*-value^†^
	WT	Het	Hom		Carrier frequency % (95% CI)	MAF % (95% CI)	MAF % (95% CI)	MAF % (95% CI)	
p.Ala531del	264	0	0	264	0	0	0	0	NA
p.Ala1622Val	264	0	0	264	0	0	0	0	NA
p.Met3891Val	264	0	0	264	0	0	0	0	NA
p.Val3933Met	264	0	0	264	0	0	0	0	NA
p.Asn3962Asp	264	0	0	264	0	0	0	0	NA
p.Cys3997Tyr	264	0	0	264	0	0	0	0	NA
p.Pro4007Arg	264	0	0	264	0	0	0	0	NA
p.Asp4013Asn	264	0	0	264	0	0	0	0	NA
p.Arg4019Cys	264	0	0	264	0	0	0	0	NA
p.Arg4062Gln	264	0	0	264	0	0	0	0	NA
p.Ile4076Val	264	0	0	264	0	0	0	0	NA
p.Arg4131Cys	264	0	0	264	0	0	0	0	NA
p.Lys4185Thr	264	0	0	264	0	0	0	0	NA
p.Gln4367Leu	264	0	0	264	0	0	0	0	NA
p.Val4567Met	264	0	0	264	0	0	0.08 (0.01–0.45)	0	NA
p.Thr4586Pro	264	0	0	264	0	0	0	0	NA
p.Pro4608Ser	264	0	0	264	0	0	0	0	NA
p.Leu4631Val	264	0	0	264	0	0	0	0	NA
p.Lys4732Thr	264	0	0	264	0	0	0	0	NA
p.Glu4750Lys	264	0	0	264	0	0	0	0	NA
p.Val4765Met	264	0	0	264	0	0	0	0	NA
**p.Arg4810Lys**	**86**	**177**	**1**	**264**	**67.42 (61.55–72.79)**	**33.90 (29.99–38.04)**	**0.80 (0.43–1.47)**	**1.05 (0.7–1.57)**	**<0.0001**
p.Asp4863Asn	264	0	0	264	0	0	0	0	NA
p.Arg4927Gln	264	0	0	264	0	0	0		NA
p.Glu4950Asp	264	0	0	264	0	0	0	0.09 (0.02–0.33)	NA
p.Ala5021Val	262	2	0	264	0.76 (0.21–2.72)	0.38 (0.10–1.37)	0.24 (0.08–0.7)	0.14 (0.05–0.41)	1.00
p.Met5136I	264	0	0	264	0	0	0	0	NA
p.Asp5160Glu	264	0	0	264	0	0	0	0	NA
p.Val5163Ile	264	0	0	264	0	0	0	0	NA
p.Glu5176Gly	264	0	0	264	0	0	0	0	NA

Abbreviations: CI, confidence interval; Het, heterozygous; Hom, homozygous; MAF, minor allele frequency; NA, not available; WT, wild type.

Significant results are shown in bold.

*Data from the Korean Reference Genome Database (http://152.99.75.168/KRGDB/)

^†^MMD patients vs. controls (*N* = 622 or *N* = 1100)

## Discussion

The major finding of this study is that the *RNF213* p.Arg4810Lys variant is the main predisposing allele for MMD in our cohort of 30 previously known mutations. Another rare variant, *RNF213* p.Ala5021Val, was identified, but did not significantly associate with MMD. Our findings are in line with previous research demonstrating that *RNF213* p.Arg4810Lys is the strongest founder variant common in East Asian MMD patients [5,6].

*RNF213* on chromosome 17q25 has been recognized as the major susceptibility gene for MMD in East Asians [5,6], and the p.Arg4810Lys variant has been identified in 95% of patients with familial MMD, as well as in 80% of patients with sporadic MMD [5]. A recent study conducted in a mouse model deficient in *RNF213* addressed the potential role of *RNF213* alteration in the development of aberrant vascular networks in chronic ischemia [12]. However, other functional missense variants of *RNF213* have been identified in both East Asians and European patients with MMD [9,10,13]. Several *RNF213* non-p.Arg4810Lys variants were recently found in Caucasian and East/South Asian cases of MMD [6,10,14,15]. Additionally, clinical manifestations and angiographic findings differ between Caucasian and East Asians [16]. The *RNF213* p.Arg4810Lys variant is reportedly associated with the ischemic-type MMD, whereas *RNF213* non-p.Arg4810Lys variants are associated with hemorrhagic-type MMD [14]. In the present study, 52.6% (139/264) of MMD patients had cerebral ischemia (TIA or cerebral infarction). Only 6.4% (17/264) of the patients had hemorrhage at diagnosis. Although the variant allele was observed at higher frequency in the ischemia group than in the hemorrhage group, this difference was not significant (35.6% and 20.6%, respectively; *P* = 0.08). Further studies of larger cohorts of Asians are required to clarify the association between the *RNF213* p.Arg4810Lys variant and clinical characteristics.

Our study revealed that ~33% of Korean MMD patients in our cohort did not harbor susceptibility variants of *RNF213*, indicating that further studies are required to discover other genetic risk factors for MMD. Several susceptibility genes have been identified for MMD: MMD-2 (gene: *RNF213*) [5,6], MMD-5 (*ACTA2*) [17], and MMD-6 with achalasia (*GUCY1A3*) [18]. Loci for the disorder have been mapped to chromosome 3p (MMD-1) [19], 8q23 (MMD-3) [20], and Xq28 (MMD-4) [21]. Further studies on other modifying factors, such as microRNAs and their polymorphisms and biomarkers (e.g., endothelial progenitor cells) are needed [22,23].

Our study was limited in that we could not determinate whether other *RNF213* rare variants apart from the 30 previously discovered *RNF213* variants were present in patients with MMD. Exome and genome sequencing could be useful tools to identify novel susceptibility genes or variants for MMD. Additionally, our study had a limited sample size. Further studies with larger cohorts including pediatric patients with MMD are needed. Lastly, intracranial stenosis can be caused by atherosclerosis or other causes (e.g., dissection) in adult patients. All patients underwent conventional angiography and high-resolution magnetic resonance imaging to preclude non-MMD pathologies in selected cases, especially when vascular studies showed controversial results in the diagnosis of MMD [24].

## Conclusions

We confirmed that, in our cohort, *RNF213* p.Arg4810Lys was strongly associated with MMD among the 30 *RNF213* variants listed in the HGMD. Our analysis also indicates that other susceptibility genes exist, providing further insight into the pathogenesis of MMD.

## Supporting information

S1 Table PCR primers used in this study for the MALDI-TOF MS genotyping. (DOCX)

## References

[1] Kuroda S, Houkin K. Moyamoya disease: current concepts and future perspectives. Lancet Neurol. 2008; 7: 1056–1066. doi: 10.1016/S1474-4422(08)70240-0 18940695 10.1016/S1474-4422(08)70240-0

[2] Baba T, Houkin K, Kuroda S. Novel epidemiological features of moyamoya disease. J Neurol Neurosurg Psychiatry. 2008; 79: 900–904. doi: 10.1136/jnnp.2007.130666 18077479 10.1136/jnnp.2007.130666

[3] Yonekawa Y, Ogata N, Kaku Y, Taub E, Imhof HG. Moyamoya disease in Europe, past and present status. Clin Neurol Neurosurg. 1997; 99 Suppl 2: S58–60. 9409407 10.1016/s0303-8467(97)00042-5

[4] Ahn IM, Park DH, Hann HJ, Kim KH, Kim HJ, Ahn HS. Incidence, prevalence, and survival of moyamoya disease in Korea: a nationwide, population-based study. Stroke. 2014; 45: 1090–1095. doi: 10.1161/STROKEAHA.113.004273 24595588 10.1161/STROKEAHA.113.004273

[5] Kamada F, Aoki Y, Narisawa A, Abe Y, Komatsuzaki S, Kikuchi A, et al A genome-wide association study identifies RNF213 as the first Moyamoya disease gene. J Hum Genet. 2011; 56: 34–40. doi: 10.1038/jhg.2010.132 21048783 10.1038/jhg.2010.132

[6] Liu W, Morito D, Takashima S, Mineharu Y, Kobayashi H, Hitomi T, et al Identification of RNF213 as a susceptibility gene for moyamoya disease and its possible role in vascular development. PLoS One. 2011; 6: e22542 doi: 10.1371/journal.pone.0022542 21799892 10.1371/journal.pone.0022542 PMC3140517

[7] Miyatake S, Miyake N, Touho H, Nishimura-Tadaki A, Kondo Y, Okada I, et al Homozygous c.14576G>A variant of RNF213 predicts early-onset and severe form of moyamoya disease. Neurology. 2012; 78: 803–810. doi: 10.1212/WNL.0b013e318249f71f 22377813 10.1212/WNL.0b013e318249f71f

[8] Mineharu Y, Takagi Y, Takahashi JC, Hashikata H, Liu W, Hitomi T, et al Rapid progression of unilateral moyamoya disease in a patient with a family history and an RNF213 risk variant. Cerebrovasc Dis. 2013; 36: 155–157. doi: 10.1159/000352065 24029639 10.1159/000352065

[9] Lee MJ, Chen YF, Fan PC, Wang KC, Wang K, Wang J, et al Mutation genotypes of RNF213 gene from moyamoya patients in Taiwan. J Neurol Sci. 2015; 353: 161–165. doi: 10.1016/j.jns.2015.04.019 25956231 10.1016/j.jns.2015.04.019

[10] Cecchi AC, Guo D, Ren Z, Flynn K, Santos-Cortez RL, Leal SM, et al RNF213 rare variants in an ethnically diverse population with Moyamoya disease. Stroke. 2014; 45: 3200–3207. doi: 10.1161/STROKEAHA.114.006244 25278557 10.1161/STROKEAHA.114.006244 PMC4420622

[11] Stenson PD, Mort M, Ball EV, Shaw K, Phillips A, Cooper DN. The Human Gene Mutation Database: building a comprehensive mutation repository for clinical and molecular genetics, diagnostic testing and personalized genomic medicine. Hum Genet. 2014; 133: 1–9. doi: 10.1007/s00439-013-1358-4 24077912 10.1007/s00439-013-1358-4 PMC3898141

[12] Ito A, Fujimura M, Niizuma K, Kanoke A, Sakata H, Morita-Fujimura Y, et al Enhanced post-ischemic angiogenesis in mice lacking RNF213; a susceptibility gene for moyamoya disease. Brain Res. 2015; 1594: 310–320. doi: 10.1016/j.brainres.2014.11.014 25446450 10.1016/j.brainres.2014.11.014

[13] Moteki Y, Onda H, Kasuya H, Yoneyama T, Okada Y, Hirota K, et al Systematic Validation of RNF213 Coding Variants in Japanese Patients With Moyamoya Disease. J Am Heart Assoc. 2015; 4 doi: 10.1161/jaha.115.001862 25964206 10.1161/JAHA.115.001862 PMC4599414

[14] Wu Z, Jiang H, Zhang L, Xu X, Zhang X, Kang Z, et al Molecular analysis of RNF213 gene for moyamoya disease in the Chinese Han population. PLoS One. 2012; 7: e48179 doi: 10.1371/journal.pone.0048179 23110205 10.1371/journal.pone.0048179 PMC3479116

[15] Ma J, Liu Y, Ma L, Huang S, Li H, You C. RNF213 polymorphism and Moyamoya disease: A systematic review and meta-analysis. Neurol India. 2013; 61: 35–39. doi: 10.4103/0028-3886.107927 23466837 10.4103/0028-3886.107927

[16] Kleinloog R, Regli L, Rinkel GJ, Klijn CJ. Regional differences in incidence and patient characteristics of moyamoya disease: a systematic review. J Neurol Neurosurg Psychiatry. 2012; 83: 531–536. doi: 10.1136/jnnp-2011-301387 22378916 10.1136/jnnp-2011-301387

[17] Guo DC, Papke CL, Tran-Fadulu V, Regalado ES, Avidan N, Johnson RJ, et al Mutations in smooth muscle alpha-actin (ACTA2) cause coronary artery disease, stroke, and Moyamoya disease, along with thoracic aortic disease. Am J Hum Genet. 2009; 84: 617–627. doi: 10.1016/j.ajhg.2009.04.007 19409525 10.1016/j.ajhg.2009.04.007 PMC2680995

[18] Herve D, Philippi A, Belbouab R, Zerah M, Chabrier S, Collardeau-Frachon S, et al Loss of alpha1beta1 soluble guanylate cyclase, the major nitric oxide receptor, leads to moyamoya and achalasia. Am J Hum Genet. 2014; 94: 385–394. doi: 10.1016/j.ajhg.2014.01.018 24581742 10.1016/j.ajhg.2014.01.018 PMC3951937

[19] Ikeda H, Sasaki T, Yoshimoto T, Fukui M, Arinami T. Mapping of a familial moyamoya disease gene to chromosome 3p24.2-p26. Am J Hum Genet. 1999; 64: 533–537. doi: 10.1086/302243 9973290 10.1086/302243 PMC1377762

[20] Sakurai K, Horiuchi Y, Ikeda H, Ikezaki K, Yoshimoto T, Fukui M, et al A novel susceptibility locus for moyamoya disease on chromosome 8q23. J Hum Genet. 2004; 49: 278–281. doi: 10.1007/s10038-004-0143-6 15362573 10.1007/s10038-004-0143-6

[21] Miskinyte S, Butler MG, Herve D, Sarret C, Nicolino M, Petralia JD, et al Loss of BRCC3 deubiquitinating enzyme leads to abnormal angiogenesis and is associated with syndromic moyamoya. Am J Hum Genet. 2011; 88: 718–728. doi: 10.1016/j.ajhg.2011.04.017 21596366 10.1016/j.ajhg.2011.04.017 PMC3113251

[22] Rafat N, Beck G, Pena-Tapia PG, Schmiedek P, Vajkoczy P. Increased levels of circulating endothelial progenitor cells in patients with Moyamoya disease. Stroke. 2009; 40: 432–438. doi: 10.1161/STROKEAHA.108.529420 19095988 10.1161/STROKEAHA.108.529420

[23] Dai D, Lu Q, Huang Q, Yang P, Hong B, Xu Y, et al Serum miRNA signature in Moyamoya disease. PLoS One. 2014; 9: e102382 doi: 10.1371/journal.pone.0102382 25093848 10.1371/journal.pone.0102382 PMC4122349

[24] Ryoo S, Cha J, Kim SJ, Choi JW, Ki CS, Kim KH, et al High-resolution magnetic resonance wall imaging findings of Moyamoya disease. Stroke. 2014; 45: 2457–2460. doi: 10.1161/STROKEAHA.114.004761 24947295 10.1161/STROKEAHA.114.004761

