# Supplementary material for: Frequency and significance of rare RNF213 variants in patients with adult moyamoya disease
Source: PLoS One. 2017 Jun 15;12(6):e0179689. doi: 10.1371/journal.pone.0179689 (PMC5472300; doi:10.1371/journal.pone.0179689)
Supplement: S1 Table — (DOCX) [file pone.0179689.s001.docx]

**Supplementary Table 1. PCR primers used in this study for the MALDI-TOF MS genotyping**

| *RNF213* variants | Forward | Reverse | Extension |
| --- | --- | --- | --- |
| p.Ala531del | ACGTTGGATGAGGATGCTGAAGGTGCTGTC | ACGTTGGATGATGAACCACATCACAGACGG | TCCAGCATGAGCGCAGC |
| p.Ala1622Val | ACGTTGGATGCAACCTCATGGTTCTGCCTC | ACGTTGGATGATATTCCCAGCAGAGTGCAG | gtggGCAGAGGCTCAGCCAGG |
| p.Met3891Val | ACGTTGGATGCTGCCTTGCATGTGCTCATC | ACGTTGGATGCGTGGCTACAGTTGGTGAAG | GAGCAGATGAGCTCCAGCGGCA |
| p.Val3933Met | ACGTTGGATGTTTTCTCCACCGCACTCTTC | ACGTTGGATGTAGTAAGAAGACGTGCTCGG | cacaGCACTCTTCGTGGAGCAC |
| p.Asn3962Asp | ACGTTGGATGATTCACAGAGAGTGCGCATC | ACGTTGGATGCCCTGCCTTTCTTCAGTGTC | CGTCTTCACGTCAGAGT |
| p.Cys3997Tyr | ACGTTGGATGTGCTTCAGAACTGATTGCCC | ACGTTGGATGGGTCCTTTGCATCTCCCAG | tcctcGGTTTGGGATTCAGCCGT |
| p.Pro4007Arg | ACGTTGGATGTCATCTGCTCTGAGGCAAAC | ACGTTGGATGCATCTGCCTGGGAGATGCAA | gggCGCAGGGCAGACAGACG |
| p.Asp4013Asn | ACGTTGGATGTCATCTGCTCTGAGGCAAAC | ACGTTGGATGTCTGCCTGGGAGATGCAAAG | CAGGCAGTGCACGTGGT |
| p.Arg4019Cys | ACGTTGGATGTCATCTGCTCTGAGGCAAAC | ACGTTGGATGTCTGCCTGGGAGATGCAAAG | caacCCAGGCCCTGAGGCAGC |
| p.Arg4062Gln | ACGTTGGATGAGCCATTGAAAAGCATGCCC | ACGTTGGATGCCTTGAAGCAAATGGTGGAC | AAAGCATGCCCGCTTCC |
| p.Ile4076Val | ACGTTGGATGAGCAGGCTCTCAATCACTTC | ACGTTGGATGGTTTCTTCGTAGACCTGGTG | GGCGGAGCGTTGTCCTTGAAGCAAA |
| p.Arg4131Cys | ACGTTGGATGTGATGTTGTGGATAAGACTC | ACGTTGGATGCTTTCCAGAAGCAAGTCTCC | gGTGGATAAGACTCCTGTCATC |
| p.Lys4185Thr | ACGTTGGATGTTCCTTGTTCCTCAGGATTC | ACGTTGGATGTCCTCTTCTAGGTGGTTCAG | TCAGGATTCAATACTTGAGA |
| p.Gln4367Leu | ACGTTGGATGTGATTTTGCAGGCCTGCAAG | ACGTTGGATGATAGCCACCTCTCTAAACAG | AAGACCCCCCAAAGCCAGC |
| p.Val4567Met | ACGTTGGATGTAAGGAGGAAGACCACTGGG | ACGTTGGATGAAGGCAGACAGAACGCAGAC | TCGGTCACATGTCACCA |
| p.Thr4586Pro | ACGTTGGATGCAGTGGTCTTCCTCCTTATC | ACGTTGGATGGGTTATACCTGGGAACTCTG | TCCTTATCCGGCTACTC |
| p.Pro4608Ser | ACGTTGGATGCAGAAAGCCTTTTGGATCCC | ACGTTGGATGCTGAACACTCTGTTCCGTTG | GCCTTTTGGATCCCTCACTG |
| p.Leu4631Val | ACGTTGGATGTGAAGGACCTGGAGCAGTTG | ACGTTGGATGTGCTCTTGGAGAAGCCTGC | GGAGCAGTTGGCCAAGATG |
| p.Lys4732Thr | ACGTTGGATGCCTGCAGCTCCAAATCTTAG | ACGTTGGATGATATATGGTGACCCAGTGAC | AGAGCAATGGACCACACTT |
| p.Glu4750Lys | ACGTTGGATGGGATGGGCACTCTTTCTTTG | ACGTTGGATGCTAAGATTTGGAGCTGCAGG | AATGTGCTGGAGGTACT |
| p.Val4765Met | ACGTTGGATGTACAGTTGAGTACCTCCAGC | ACGTTGGATGTTCTGCAGGAAATGCCAGAG | AGAAAAATGGCAAAGAAAGA |
| p.Arg4810Lys | ACGTTGGATGAGTGAAGCAGTTCCAGAACG | ACGTTGGATGACCTGAGCTGTGCTTGCTGA | TTGAATACAGCTCCATCA |
| p.Asp4863Asn | ACGTTGGATGAAGGTGAGATCAACCTACCC | ACGTTGGATGGTGGCAAGAGGATCTCAAAC | gggGCACTGACTTGGATCTG |
| p.Arg4927Gln | ACGTTGGATGTGGAGAGAATCAGTGGAGTC | ACGTTGGATGTTTCTGCCCCTCAGCTATTC | AATCAGTGGAGTCAGGTCC |
| p.Glu4950Asp | ACGTTGGATGACTGATTCTCTCCAACTGCC | ACGTTGGATGATCTGCCGCTGAATCTTCTC | AGAGAGACCGTGCAGGA |
| p.Ala5021Val | ACGTTGGATGGAGTGACTTCTACGACAGAC | ACGTTGGATGTCGGTCATTAGTGCCATCAG | CTACGACAGACAGCACTTCACAG |
| p.Met5136I | ACGTTGGATGTCCTCGGTTTGGGTTTGGG | ACGTTGGATGAAATCAGACTGGCCTAGACG | GGGTTCTTTAGTTTCAAGATTAT |
| p.Asp5160Glu | ACGTTGGATGCTCTTCTTTGGTTTTTCAGCC | ACGTTGGATGGGGAACTGAGATGCCATTTC | GGTTTTTCAGCCTGAGAGA |
| p.Val5163Ile | ACGTTGGATGGGGAACTGAGATGCCATTTC | ACGTTGGATGTTGGTTTTTCAGCCTGAGAG | CTTTCTTTAGTTTGCATGTAACTTA |
| p.Glu5176Gly | ACGTTGGATGGCAGTATCTCTTCTGGGAAC | ACGTTGGATGCTGAGAGACACTCTCGTAAG | GGAACTGAGATGCCATT |

Abbreviation: MALDI-TOF MS, matrix-assisted laser desorption/ionization time-of-flight mass spectrometry.

*RNF213* reference accession number: NM_001256071.1
